# Coumarin derivatives as new anti-biofilm agents against *Staphylococcus aureus*

**DOI:** 10.1371/journal.pone.0307439

**Published:** 2024-09-19

**Authors:** Atia-tul- Wahab, Faiza Nadeem, Uzma Salar, Hafiz Muhammad Bilal, Mehak Farooqui, Sumaira Javaid, Sohira Sadaf, Khalid M. Khan, M. Iqbal Choudhary

**Affiliations:** 1 Dr. Panjwani Center for Molecular Medicine and Drug Research, International Center for Chemical and Biological Sciences, University of Karachi, Karachi, Pakistan; 2 H. E. J. Research Institute of Chemistry, International Center for Chemical and Biological Sciences, University of Karachi, Karachi, Pakistan; 3 Department of Biochemistry, Faculty of Science, King Abdulaziz University, Jeddah, Saudi Arabia; Qassim University, SAUDI ARABIA

## Abstract

*Staphylococcus aureus* infections are the primary causes of morbidity, and mortality, particularly in immuno-compromised individuals. *S*. *aureus* associated infections are acquired from community, as well as hospital settings, and difficult to treat because of the emerging resistance against available antibiotics. One of the key factors of its resistance is the biofilm formation, which can be targeted to treat *S*. *aureus-*induced infections. Currently, there is no drug available that function by targeting the biofilm. This unmet need demands the discovery of drug candidates against *S*. *aureus* biofilm. The present study was designed to evaluate coumarin derivatives **1**–**21** against *S*. *aureus* biofilm. The 96-well plate crystal violet assay was employed for the quantification of biofilm. Results showed that the coumarin derivatives **2**–**4**, **10**, and **17** possess potent antibiofilm activity, with MBIC values between 25–100 μg/mL. The results were further confirmed through atomic force microscopy (AFM), scanning electron (SEM), and fluorescence microscopic studies. The quantitative RT-PCR analysis revealed the downregulation of biofilm associated genes, *icaA* and *icaD*. These coumarin derivatives were also found to be non-cytotoxic to fibroblasts. This study, therefore, identifies the antibiofilm potential of coumarin derivatives that will pave the way for further research on these derivatives.

## Introduction

*Staphylococcus aureus* is an opportunistic pathogen, usually inhabits nasopharynx, and skin. It causes common nosocomial infections, including pneumonia, life-threatening endocarditis, necrotizing fasciitis, infections of the heart valves, septicemia, and skin structure infections. It can also infect surgical wounds, and lower respiratory tract [1, 2]. The ability of *S*. *aureus* to cause diverse recalcitrant infections is mainly due to capacity to resist against multiple drugs, such as vancomycin, methicillin, and daptomycin. Its diverse virulence factors, and ability to produce biofilm have been the major hurdle in the development of new drugs [3].

A biofilm is an immobilized microbial community in which cells are firmly encased in a protective extracellular polymeric substances (EPS) matrix, and adhered either to a surface or to other cells. It cannot be removed by gentle rinsing, as in addition to cells, it also consists of mineral crystals, silt, and corrosion particles [1]. Biofilm forming property of *S*. *aureus* has remarkable consequences in the medical field [4]). Such microorganisms can produce toxins (like hemolysin, *α*-toxin, enterotoxins, TSST-1, coagulase, and protein A) which further aids in biofilm formation [5]. Process of biofilm formation includes the involvement of a variety of genes and proteins, such as protease, DNase, D-amino acids, *c*-di-GMP, *cis*-2-decenoic acid, phenol-soluble polypeptides, environmental factors like pH, and quorum sensing mechanisms. Biofilms are typically formed through a two-step process. Initially, the cells bind to the surface by means of capsular antigen, specifically capsular polysaccharide/adhesion (PS/A), followed by multiplication of bacterial cells to form a multilayered biofilm, accompanied with the secretion of polysaccharide intercellular adhesion (PIA) molecules [5].

The two main pathways that cause biofilm-mediated resistance are as follows. First, the biofilm matrix itself restricts chemotherapeutics from reaching their targets by limiting diffusion or repulsion [6–9]. The second mechanism is that, in contrast to planktonic bacteria, the physiology of biofilm-dwelling bacteria is altered [10, 11]. Therefore, targeting biofilm is a promising approach to treat infections caused by *S*. *aureus*, and other biofilm forming microorganisms. Currently, there is no drug available for clinical use that functions by targeting biofilm formation. However, scientists have identified several lysins, and small molecular compounds as potent inhibitors of biofilm forming MRSA strains *in vitro* and *in vivo* [5, 7, 12, 13]. Discovery of anti-biofilm compounds, capable of treating biofilm related infections, is an unmet need of public health.

The current study was, therefore, designed to evaluate several classes of compounds of synthetic origin for their ability to interfere with biofilm formation. We have found derivatives of coumarin scaffold exhibit the anti-biofilm activity, as presented in the Table 1. Coumarins form an important class known for broad biological activities, such as anti-oxidant, anti-cancer, anti-microbial, and anti-quorum sensing properties [14, 15].

**Table 1:** List of ATCC strains used for the evaluation of biofilm formation.

S. No	Bacterial Strain	Genetic Profile	Biofilm Phenotype
1.	ATCC 700699	Resistant	Weak
2.	ATCC 43300	Resistant	Moderate
3.	ATCC 6538	Resistant	Strong
4.	ATCC 25923	Sensitive	Moderate

## Materials and methods

### Synthesis of compounds

Synthesis of hydrazinyl thiazole substituted coumarin analog **1–21** were performed, as described previously by Salar *et al*. [15, 16].

### Materials

Brain heart infusion broth (Thermo Fisher Scientific, UK), Baird Parker agar (Thermo Fisher Scientific, UK), crystal violet (Sigma Aldrich, USA), acetic acid and glucose (DAE JUNG, Korea), dimethylsulfoxide (DMSO; Merck, Germany), phosphate buffer saline (PBS, Thermo Fisher, UK), Triton X (Sigma Aldrich, USA), Trypticase Soya broth (Merck, Germany), SYTO-9 (Thermo Scientific, UK), propidium iodide (Sigma Aldrich, USA), Dulbecco’s modified eagle medium (DMEM, Sigma Aldrich, USA), and Fetal Bovine Albumin serum (FBS, Sigma Aldrich, USA).

### Culturing of bacterial strain and growth conditions

*S*. *aureus* strains were acquired from American Type Culture Collection (ATCC 6538, ATCC 700699, ATCC 43300, and ATCC 25923), USA. Initially the strains were inoculated in trypticase soya broth overnight, followed by culturing of into Baird Parker agar plate for 48 h at 37°C. Single colony was then picked, and inoculated into brain heart infusion (BHI) broth. The cultures were then preserved in 10% glycerol in BHI, and kept at -80°C, until required for the experiments.

### Evaluation of biofilm inhibitory potential of coumarin compounds

All the strains of *S*. *aureus* were evaluated to check their biofilm forming potential (Table 1). Among four fully characterized strains, ATCC 6538 was found to have the ability to form high biofilm content between other genetically resistant strains [17, 18].

The quantitative determination of biofilm inhibition was carried out by the crystal violet assay on a sterile 96-well plate [19, 20] with slight modifications. The glycerol stock of *S*. *aureus* was revived overnight at 37°C at 80 rpm. The overnight culture was then diluted 100 folds in fresh BHI broth, supplemented with 0.25% glucose. The cell density was adjusted to 1×10^7^ and 195μL of the cell suspension was added in 96-well plate.

The test compounds (100 μg/mL) were then added to each well, and plate was incubated at 37°C for 24 hours. Following that, the plate was washed with sterile distilled water in order to decant the non-adherent cells, and media. The plate was then dried at 60°C for 1 hour, and 200 μL of 0.1% (w/v) crystal violet was added to stain biofilm for 20 minutes. The plate was washed again, air dried, and dispensed 30% (v/v) acetic acid in each well to dissolve the biofilm. The inhibition of biofilm formation was analyzed by measuring the absorbance at 595 nm using the microplate reader (Multiskan Sky, Thermo Scientific, USA).

The percent inhibition of biofilm formation was calculated *via* the following formula:

$$100-\left(ƛ\;Treated-ƛ\;Blank/\;ƛ\;Control-ƛ\;Blank\right)\times 100$$

Whereƛ=absorbance

### Determination of Minimum Biofilm Inhibitory Concentration (MBIC)

For the determination of minimum biofilm inhibitory concentration (MBIC), compounds with antibiofilm activity were two-fold diluted serially from concentration of 100 μg/mL to 3.125 μg/mL, and processed for antibiofilm activity determination, as described earlier [21].

### Atomic force microscopy analysis

The size and structure of disrupted biofilm was analyzed [22]. Briefly, a pure single colony of *S*. *aureus* was inoculated in BHI broth and grown for 18–24 hours at 37°C. Following incubation, the cells were diluted in BHI broth, and density was adjusted to 1×10^7^ before loading on a sterile silicon chip placed in 24-well plate, then silicon chips were treated with active compounds. The plate was then sealed, and placed in an incubator for 24 hours at 37°C. After incubation, the treated silicon chips were washed with autoclaved distilled water thrice, air dried, and the samples were analyzed by atomic force microscope (Agilent Technologies, USA).

### Biofilm analysis by fluorescence microscopy

Briefly, 1×10^7^ cells were loaded in 96-well plate, and treated with active compounds. Plate was sealed, and incubated for 24 hours at 37°C. Following incubation, plate was washed with sterile distilled water, and cells were stained with 1 mM SYTO-9 and 5 μM PI, for 30 minutes in dark at room temperature. After incubation, the wells were washed again, and air dried. Images were taken, and then visualized by Nikon Eclipse TE2000, USA [23]. Propidium Iodide (PI) staining indicates membrane integrity, as it can only pass through compromised membranes of non-viable cells, giving red fluorescence. On the contrary, Syto-9 can pass through all cells despite of their membrane integrity, and binds to DNA/RNA by emitting green fluorescence.

### Evaluating morphology by scanning electron microscopy

A single isolated colony of *S*. *aureus* was inoculated in BHI, and kept at 37°C for 17–18 hours. Cell density was adjusted to 1×10^7^ cells. Active compounds were added in different concentrations. The suspension was added to glass slides (Carl Roth®) and incubated at 37°C for 24 hours. After incubation, the slides were washed and adherent cells were fixed with 5% glutaraldehyde for 1 hour. The samples were then dehydrated in graded ethanol 30, 50, 70, 80, 90, and 100% for 15 minutes each [24, 25]. The samples were analyzed by Scanning Electron Microscope (Apreo 2 C Lo Vac, Thermo Fisher Scientific, USA).

### RNA extraction, cDNA synthesis, and RT-PCR

1×10^7^ bacterial cells were inoculated with test compound treated and untreated controls in a 96-well plate as mentioned above. Total RNA was isolated using the RiboPure™-Bacteria kit (Thermo Scientific, USA), following the manufacturer’s protocol. Quality of RNA was assessed using the nanodrop. RevertAid Frist Strand cDNA synthesis kit (Thermo Fisher Scientific, USA) was used to synthesize complementary DNA (cDNA), as per the manufacturer’s protocol. The cDNA synthesis process was started for 5 minutes at 25°C, followed by 60 min incubation at 45°C. The reaction was terminated at 70°C for 5 min. The PCR process was then started by preparing a reaction mixture of 10 μL by adding 1 μL of cDNA, 1 μL of forward, and reverse primers, 3 μL of the Maxima SYBR Green/ROX qPCR Master Mix (2X) kit (Thermo Scientific, USA), and 5 μL of nuclease free water in PCR strips (Table 2). The program consisted of initial denaturation at 95°C for 10 minutes, followed by 40 cycles of denaturation at 95°C for 15 seconds, annealing at 60°C for 60 seconds, and extension at 72°C for 20 seconds. The data were analyzed by using the CFX Manager Software from (BIO-RAD, USA). The expression levels of the target genes were normalized to the expression of housekeeping gene (16S rRNA), and calculated using the ΔΔCt method [26, 27].

**Table 2:** List of primers used for gene expression analysis.

Genes	Primer	Sequence (5’-3’)
*16S rRNA*	Forward	5ʹ-GTAGGTGGCAAGCGTTATCC-3ʹ
	Reverse	5ʹ-CGCACATCAGCGTCAG-3ʹ
*icaD*	Forward	5ʹ-ATGGTCAAGCCCAGACAGAG-3ʹ
	Reverse	5ʹ-AGTATTTTCAATGTTTAAAGCAA-3ʹ
*icaA*	Forward	5ʹ-ACACTTGCTGGCGCAGTCAA-3ʹ
	Reverse	5ʹ-TCTGGAACCAACATCCAACA-3ʹ
*fnbA*	Forward	5ʹ-AAATTGGGAGCAGCATCAGT-3ʹ
	Reverse	5ʹ-GCAGCTGAATTCCCATTTTC-3ʹ

### Cytotoxicity assay

Cytotoxic effect of the active compounds was determined by using MTT (3- [4, 5-dimethylthiazole-2-yl]-2, 5-diphenyl-tetrazolium bromide) assay [28, 29]. Briefly, BJ human fibroblast cells (CRL 2522) were cultured in DMEM media, supplemented with 10% FBS, 1% pen/strep, and 2 mM L-glutamine and incubated in 5% CO_2_ at 37°C in a humidified incubator. Exponentially growing cells were trypsinized upon 80% confluence, and 9x10^3^ cells/ well were introduced in 96-well plate. Following overnight incubation, the media was removed, and fresh media was added with different concentrations of test compounds ranging from 0.5–15 μg/mL. The plate was incubated for 48 hours at 37°C. Following incubation, MTT (0.5 mg/mL) was introduced to each well, and then plate was further incubated for 3 hours. After that, the dye was removed, and formazan crystals were solubilized in DMSO. The degree of MTT reduction to formazan was calculated by measuring the absorbance at 550nm, using a microplate reader (Spectra Maxplus, Molecular Devices, USA) [29, 30].

The percent inhibition was calculated *via* given formula:

$$\%\;Inhibition\;=100-\left(ƛ\;Treated-ƛ\;Negative\;Control/\;ƛ\;Positive\;Control-ƛ\;Negative\;Control\right)\times 100$$

## Results

### Screening of coumarin derivatives for antibacterial and antibiofilm activities

Evaluation of anti-biofilm activity is mostly compared with the untreated reference growth control. Biofilm measured at the same endpoint as the treated biofilm. Results indicated that the coumarin derivatives **2**–**4**, **10**, and **17** inhibit the formation of biofilm (≥50% inhibition) with low inhibition of bacterial growth *i*.*e*., ≤ 30% (Table 3).

**Table 3:** Antibacterial and antibiofilm activity of various coumarin derivatives at 100 μg/mL against biofilm of *S*. *aureus*.

Compound	IUPAC names	Antibacterial Activity (% Inhibition ± SEM)	Antibiofilm Activity (% Inhibition ± SEM)	MBIC (μg/mL)
**1**	*(E*)-2-((4-Chloro-6-methyl-2-oxo-2*H*-chromen-3-yl)methylene)hydrazinecarbothioamide	NA^a^	NA^a^	ND^b^
**2**	(*E*)-4-Chloro-6-methyl-3-((2-(4-(3-nitrophenyl)thiazol-2-yl)hydrazono)methyl)-2*H*-chromen-2-one	15 ± 4.53	90 ± 3.24	100
**3**	(*E*)-4-(2-(2-((4-Chloro-6-methyl-2-oxo-2*H*-chromen-3-yl)methylene)hydrazinyl)thiazol-4-yl)benzonitrile	27 ± 6.41	91 ± 6.31	25
**4**	(*E*)-4-Chloro-6-methyl-3-((2-(4-(4-nitrophenyl)thiazol-2-yl)hydrazono)methyl)-2*H*-chromen-2-one	21 ± 4.51	90 ± 9.30	50
**5**	(*E*)-4-Chloro-3-((2-(4-(2,4-dichlorophenyl)thiazol-2-yl)hydrazono)methyl)-6-methyl- 2*H*-chromen-2-one	36 ± 8.77	No Inhibition	ND^b^
**6**	(*E*)-3-((2-(4-(4-Bromophenyl)thiazol-2-yl)hydrazono)methyl)-4-chloro-6-methyl-2*H*- chromen-2-one	72 ± 7.22	61 ± 4.05	ND^b^
**7**	*(E*)-4-Chloro-3-((2-(4-(4-chlorophenyl)thiazol-2-yl)hydrazono)methyl)-6-methyl-2*H*- chromen-2-one	41 ± 11.1	No Inhibition	ND^b^
**8**	(*E*)-4-Chloro-3-((2-(4-(4-methoxyphenyl)thiazol-2-yl)hydrazono)methyl)-6-methyl-2*H*- chromen-2-one	52 ± 5.08	66 ± 5.11	ND^b^
**9**	(*E*)-2-(1-(2-Oxo-2*H*-chromen-3-yl)ethylidene)hydrazinecarbothioamide	55 ± 5.43	No Inhibition	ND^b^
**10**	(*E*)-3-(1-(2-(4-(4-Bromophenyl)thiazol-2-yl)hydrazono)ethyl)-2*H*-chromen-2-one	18 ± 3.73	69 ± 9.34	100
**11**	(*E*)-3-(1-(2-(4-(4-Chlorophenyl)thiazol-2-yl)hydrazono)ethyl)-2*H*-chromen-2-one	88 ± 4.32	70 ± 7.09	ND^b^
**12**	(*E*)-3-(1-(2-(4-(3,4-Dichlorophenyl)thiazol-2-yl)hydrazono)ethyl)-2*H*-chromen-2-one	NA^b^	No Inhibition	ND^b^
**13**	(*E*)-3-(1-(2-(4-(3-Nitrophenyl)thiazol-2-yl) hydrazono)ethyl)-2*H*-chromen-2-one	No Inhibition	No Inhibition	ND^b^
**14**	(*E*)-4-(2-(2-(1-(2-Oxo-2*H*-chromen-3-yl)ethylidene)hydrazinyl)thiazol-4-yl)benzonitrile	61 ± 9.33	68 ± 6.63	ND^b^
**15**	(*E*)-3-(1-(2-(4-(2-Hydroxyphenyl)thiazol-2-yl)hydrazono)ethyl)-2*H*-chromen-2-one	73 ± 7.16	34 ± 5.11	ND^b^
**16**	(*E*)-3-(2-(2-Benzylidenehydrazinyl)thiazol-4-yl)-2*H*-chromen-2-one	NA^b^	No inhibition	ND^b^
**17**	(*E*)-3-(2-(2-(3-Bromo-5- chlorobenzylidene)hydrazinyl)thiazol-4-yl)-2*H*-chromen-2-one	20 ± 4.64	69 ± 1.75	100
**18**	(*E*)-3-(2-(2-(3-Chlorobenzylidene)hydrazinyl)thiazol-4-yl)-2*H*-chromen-2-one	32 ± 8.97	25 ± 5.26	ND^b^
**19**	(*E*)-3-(2-(2-(4-Chlorobenzylidene)hydrazinyl)thiazol-4-yl)-2*H*-chromen-2-one	No Inhibition	37 ± 6.98	ND^b^
**20**	(*E*)-3-(2-(2-(2,4-Dichlorobenzylidene)hydrazinyl)thiazol-4-yl)-2*H*-chromen-2-one	NA^b^	No Inhibition	ND^b^
**21**	(*E*)-3-(2-(2-(2-Nitrobenzylidene)hydrazinyl)thiazol-4-yl)-2*H*-chromen-2-one	NA^b^	No Inhibition	ND^b^

NA^a^: Not Active, ND^b^: Not Determined

Parent compound **1**, a hydrazinyl thiazole substituted coumarin analog, was found inactive in inhibiting the bacterial cell growth and biofilm formation at 100 μg/mL, while compound **2** with 3-nitrophenyl substitution on hydrazinyl thiazole moiety caused 90% inhibition of biofilm formation, and 15% bacterial growth inhibition at 100 μg/mL. Compound **3** substituted with 4-benzonitrile moiety, caused 91% inhibition of biofilm formation with 27% growth inhibition. Compound **4**, with 4-nitrophenyl substitution, also exhibited biofilm inhibition at 100 μg/mL (90%) similar to compound **2**, indicating that nitro group has no influence on increasing or reducing the biofilm formation inhibition. Compounds **6**, and **8** with substitutions of either halogens (bromo) or methoxy groups induced biofilm inhibition. However, their ability to inhibit the growth of bacteria was more than their anti-biofilm activity at 100 μg/mL (Table 3). Compound **7** with substitutions of halogens (chloro) was unable to inhibit the bacterial growth and biofilm formation. Compound **9** caused 55% inhibition of bacterial growth and 22% biofilm inhibition, while compounds **11**, **14**, and **15** with substitutions of either halogens, hydroxyl, or cyano groups induced biofilm inhibition, however their ability to inhibit the growth of bacteria was more than their anti-biofilm activity at 100 μg/mL (Table 3). Compounds **10** (with 4-bromophenyl group) and **17** (with 3-bromo, 5-chloro substituted phenyl group) both induced 69% anti-biofilm activity with 18 and 20% growth inhibition, respectively, at 100 μg/mL. Compounds **12**, **13**, and **16**–**21** were unable to inhibit the growth of bacteria, as well as biofilm formation.

Based on the results, we propose that substitution of halogen, nitro, cyanide, and methoxy groups on phenyl ring has a significant influence on the anti-biofilm activity. In addition to this, number of halogen groups along with their position on phenyl ring also influenced the biofilm formation ability of *S*. *aureus*.

### Determination of Minimum Biofilm Inhibitory Concentration (MBIC)

**Fig 1:**
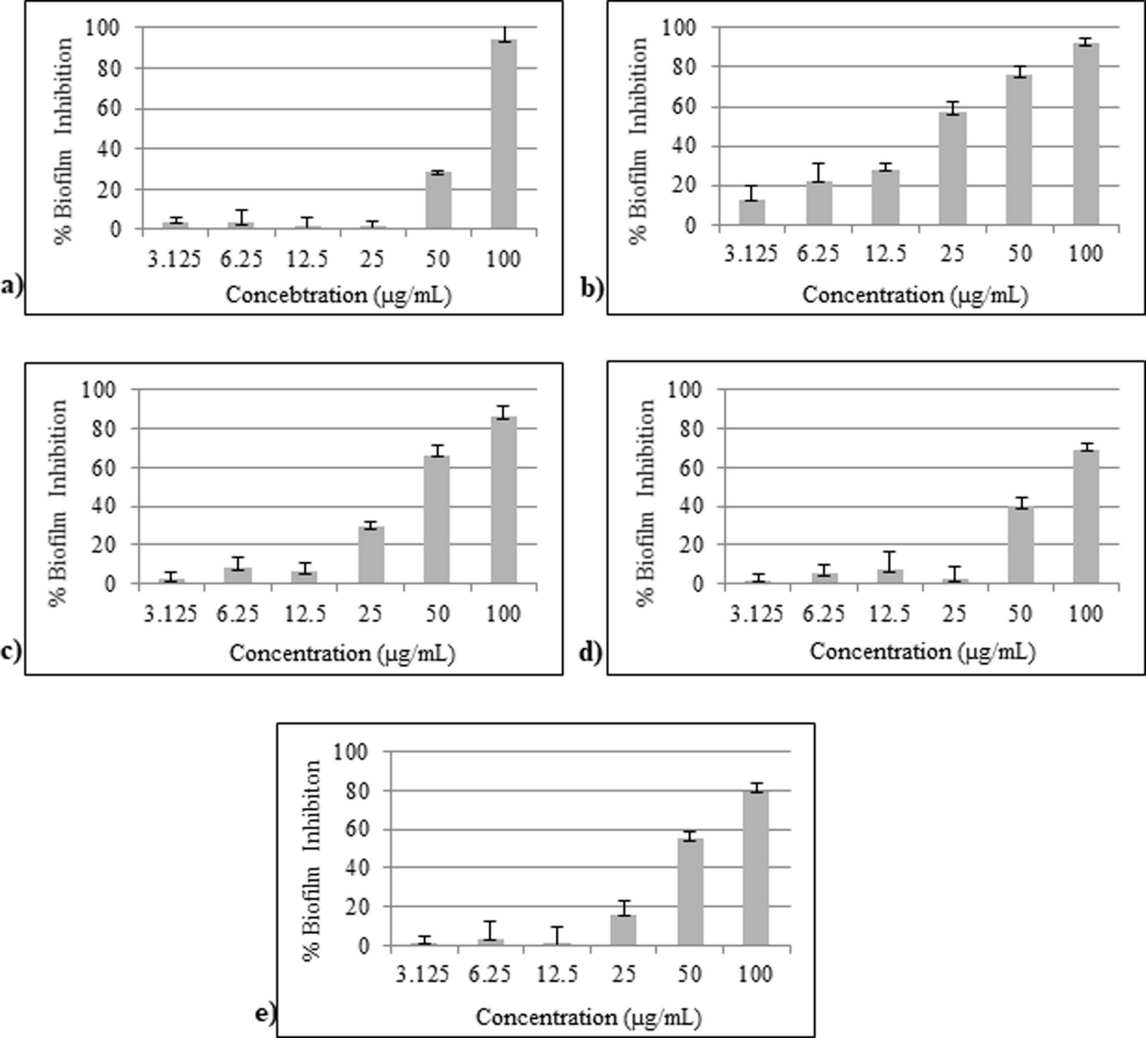
Graphical representations of MBIC values of potent coumarin derivatives against *S*. *aureus*. (a) MBIC value (100 μg/mL) of compound **2** (b) MBIC value (25 μg/mL) of compound **3** (c) MBIC value (50 μg/mL) of compound **4** (d) MBIC value (100 μg/mL) of compound **10** (e) MBIC value (100 μg/mL) of compound **17**.

Compounds **2**, **3**, **4**, **10**, and **17** with significant antibiofilm activity and low growth inhibitory potential were selected for the determination of Minimum Biofilm Inhibitory Concentration (MBICs). The MBIC values for compounds **2**, **10**, and **17** were found to be active at 100 μg/mL, while compounds **3**, and **4** showed a MBIC of 25, and 50 μg/mL, respectively (Table 3 and Fig 1).

### Analysis of biofilm disruption and membrane roughness by Atomic Force Microscopy (AFM)

Compounds with a significant anti-biofilm activity, and less growth inhibitory potential, were evaluated for their ability to disrupt already formed biofilm *via* AFM studies. This technique provides information about the 3D surface topography, cellular morphology, and biofilm disruption [31]. The AFM images provide quantitative analysis of biofilm biomass in terms of thickness, and EPS matrix level [32].

**Fig 2:**
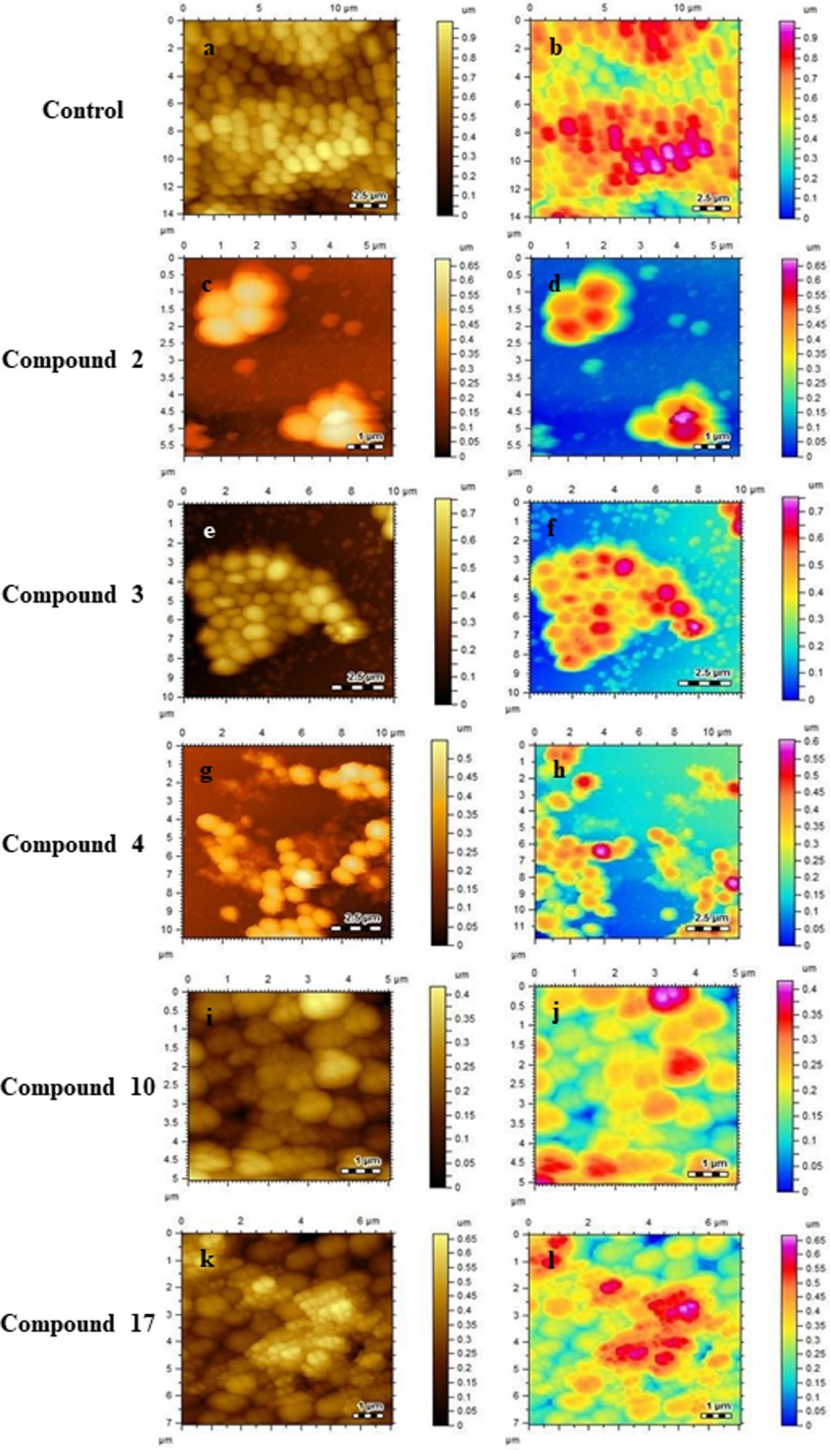
(a-b) represents two-dimensional AFM images, and surface profiles of control biofilm (c-l) depicts 2-D images of biofilm after exposure with coumarin derivatives **2, 3, 4, 10,** and **17** respectively.

The exterior of control biofilm cells featured compact, smooth cell surfaces, a uniform outer membrane with an inflated appearance, and a regular biofilm divisional pattern. Additionally, it was apparent that the maximal EPS matrix maintained structural integrity. There were no apparent pores, grooves, cell membrane ruptures, or bubbles in the control cells. Thus, these images visibly confirmed the preserved integrity of the attached biofilms (Fig 2A and 2B).

Compounds **2**, **3**, and **4** with MBIC values of 100, 25, and 50 μg/mL, respectively, were found to potentially disrupt and degrade biofilm to varying degrees, as compared to control *S*. *aureus* cells (Fig 2C–2H). On the surface, cellular debris was identified. There was also blebbing, cellular collapse with substantial bulges, grooves, and lysis. The cells were seen to be swollen with increased surface area. Additionally, compound **2** demonstrated island-like cell clusters on the surface, indicating increased biofilm disruption in contrast to the smooth, intact, multilayered biofilm matrix, exhibited by control biofilms. Disrupted biofilm surfaces were also observed when biofilm forming cells were treated with compounds **17**, and **10**, having MBIC value of 100 μg/mL, respectively (Fig 2I and 2J). In addition to this, deep ruptures and roughness on biofilm surface, along with leakage of intracellular material and EPS matrix, were also observed.

### Assessment of bacterial viability through fluorescence microscopy

The effects of compounds **2**, **3**, **4**, **10**, and **17** on biofilm were further investigated *via* fluorescence microscopy to obtain direct morphological information about cells [33]. The live and dead cells of biofilm cells of the *S*. *aureus* strain (ATCC 6538) were investigated by Syto9 and counterstained by propidium iodide (PI), respectively [32] and observed under fluorescence microscope. The biofilm of *S*. *aureus* cells without any treatment has served as respective control.

**Fig 3:**
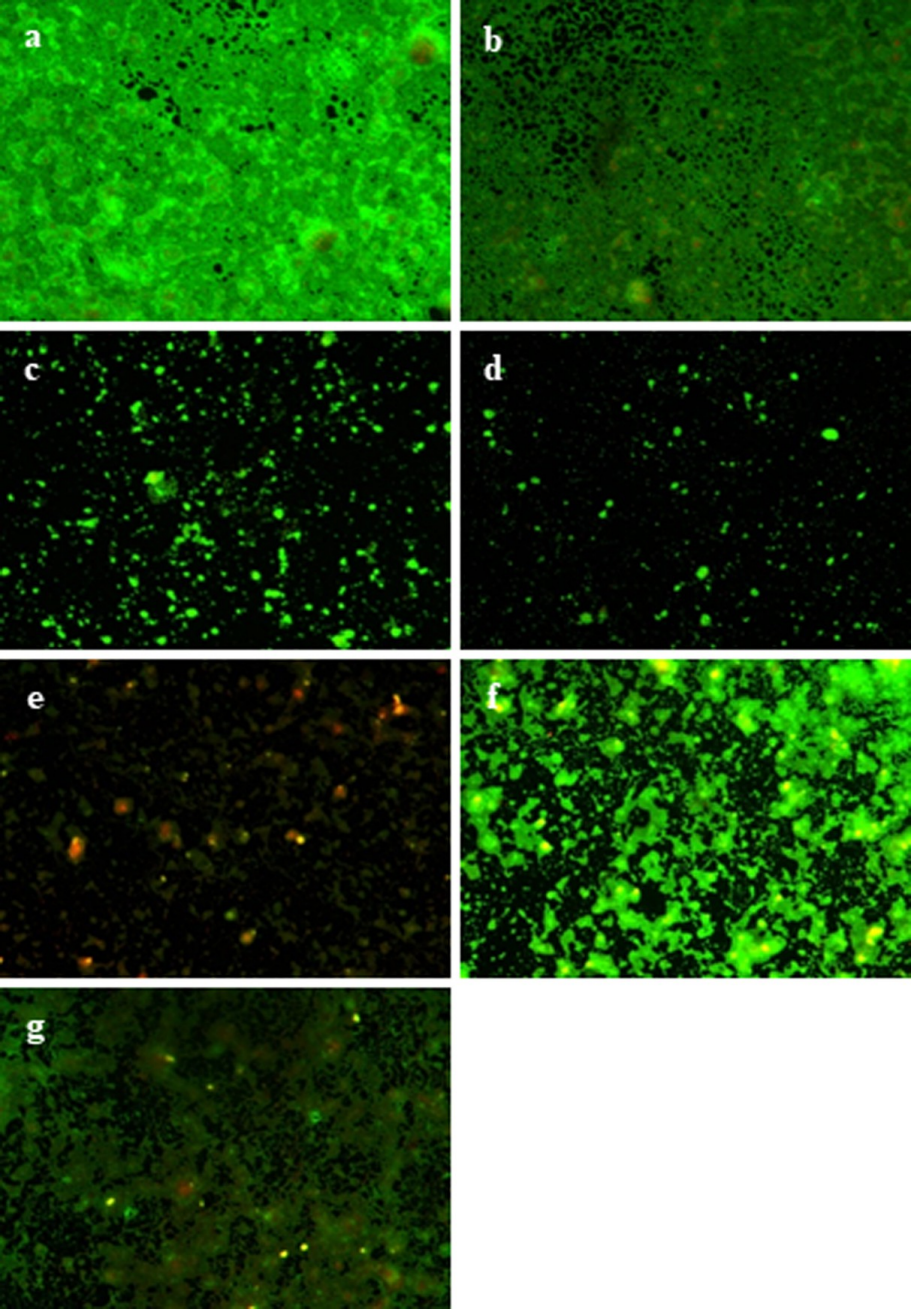
Fluorescence microscopic images of untreated control of *S*. *aureus* (6538) shows normal and regular biofilm matrix (a, b) when stained with SYTO-9. Image (c-g) depicts biofilm treated with coumarin derivatives **2**–**4**, **10**, and **17**, respectively. The images showed biofilm exhibiting cellular morphology alterations with reduced biofilm formation.

Mature biofilms were formed by *S*. *aureus* cells adhering to surface, continuing to attach, and divide. Within 24 hours, *S*. *aureus* produced significantly large aggregates on submerged surfaces that are indicative of mature biofilms. Compounds **3** and **4** with MBIC of 25 and 50 μg/mL, respectively, caused a mild to extensive biofilm disruption and degradation, as shown in Fig 3A, 3B, 3D and 3E). Disruption on the surface of biofilm was also observed on treatment with compounds **2**, **10**, and **17** with MBIC value of 100 μg/mL (Fig 3C, 3F and 3G). Loss of regular cellular morphology, leakage of intracellular material, and EPS matrix was also observed. In addition to this, scattered cellular arrangement, the decrease total biomass and average thickness of the biofilm was also confirmed by fluorescence microscopic analysis.

### Tracking alternations in cellular morphology using scanning electron microscopy

**Fig 4:**
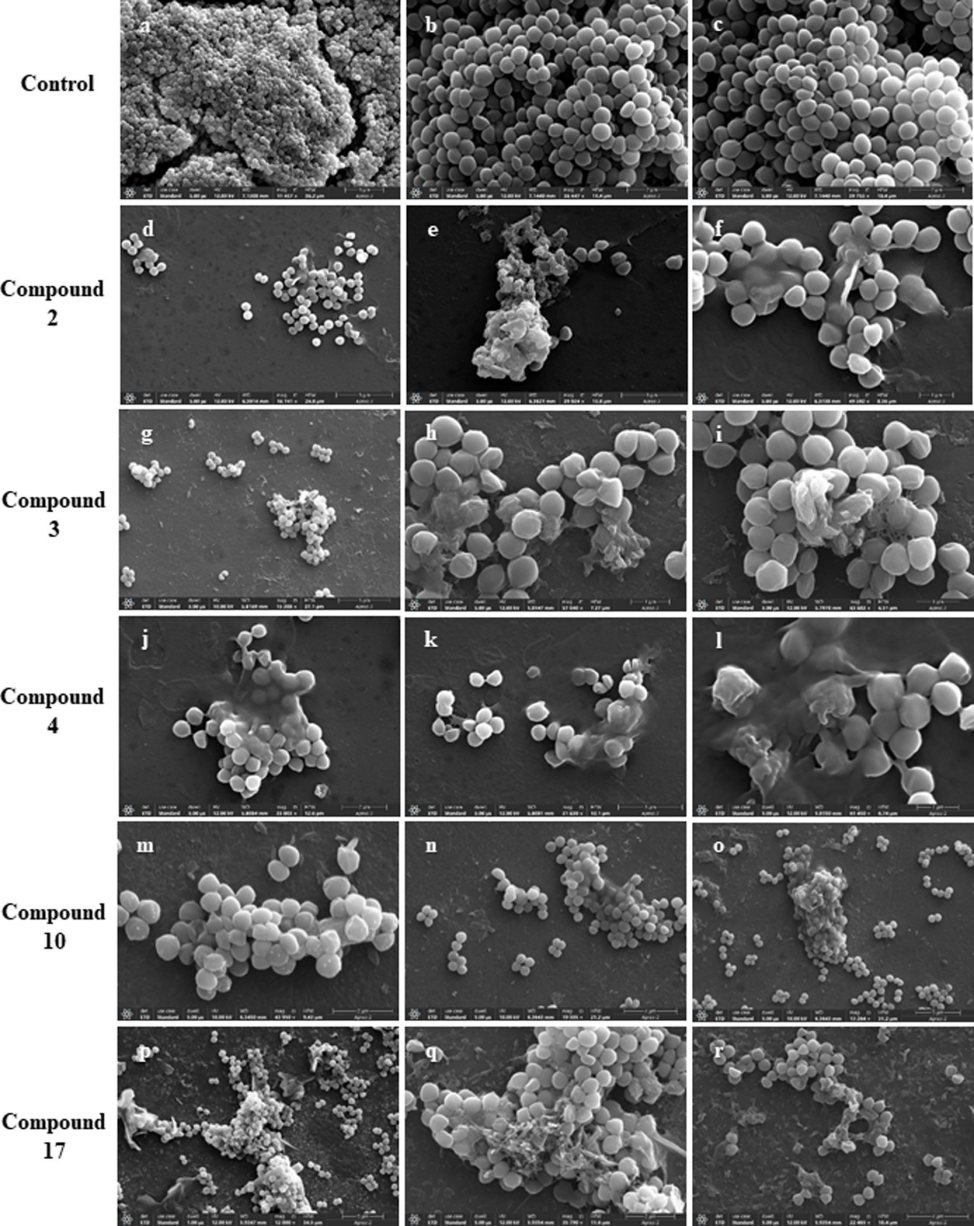
Scanning Electron Micrograph of untreated biofilm cells of *S*. *aureus* (6538) showing clusters of cells joined together in biofilm (a, b, c). Image (d-f) depicts biofilm treated with compound **2** at 100 μg/mL. Image (g-i) have been treated with 25 μg/ml of compound **3**. Image (j-k) illustrates the effect of compound **4** at 50 μg/mL. Similarly, Image (m-r) show the effect of compounds **10** and **17** on biofilm of *S*. *aureus* at 100 μg/mL respectively.

In order to gain a better sight into the process of biofilm formation and to have an understanding about the disruption mechanism of the active compounds, SEM analysis was performed. In comparison to the spherical, grape like arranged clusters of *S*. *aureus* in the control, as shown in Fig 4A–4C) while the treated cells shown in Fig 4D–4R were disoriented showing leaking of intracellular material resulting in cell shrinking. The reduction in biofilm mass can also be clearly seen in the treated samples. The irregularly deposited cells were found to have been enclosed in a matrix while some of the cells seemed to have completely lost their morphology. The ability of the cells to compact or bind was also greatly reduced.

### Transcriptional changes induced in biofilm-related genes

**Fig 5:**
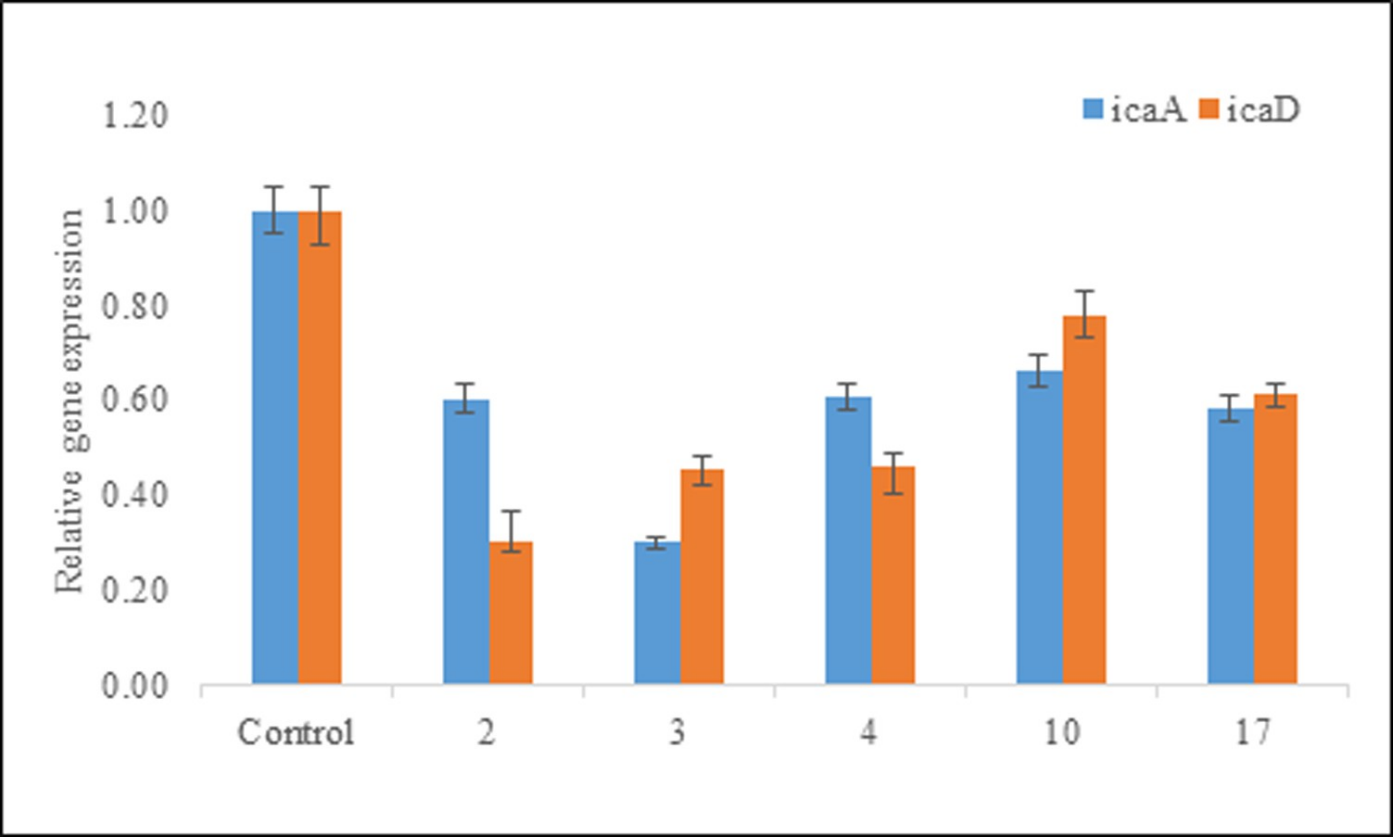
Comparative gene expression level of biofilm associated genes. As determined by real-time PCR, the graphs represent the fold change in gene expression between treated and untreated cells. After treatment with compounds **2**–**4**, **10**, and **17**, *S*. *aureus* (ATCC 6538) exhibits a down-regulation of the quantitative relative expression of the intercellular adhesion genes (*icaA* and *icaD*).

The expression levels of intercellular adhesion genes (*icaA* and *icaD*) and surface-anchored fibronectin-binding protien (*fnbA*) were analyzed in treated and untreated samples and compared to evaluate the inhibitory effect of coumarin derivatives on biofilm formation. Cells were treated with active compounds at their respective MBIC values. Noticeably, both the biofilm associated genes, *icaA* and *icaD*, were found to be downregulated, as compared to untreated control (Fig 5). However, the *fnbA* gene remained unaffected. Compounds **2–4**, **10**, and **17** downregulated the *icaA* and *icaD* genes but did not affect the expression of housekeeping gene (16S rRNA). However, the reason that why all the coumarin derivatives were found ineffective on *fnbA* remains unclear. Moreover, it can also be stated that biofilm formation is a multifactorial process involving several genes including *SarA* and agr (accessory gene regulator).

### Analysis of cytotoxicity by MTT assay

All compounds were found non-cytotoxic towards the human fibroblast BJ cells (CRL2522), indicating that they can be evaluated safely for further mechanistic, and *in-vivo* studies.

## Discussion

The potential of bacterial pathogens to develop resistance against the first line of antibiotic classes has endangered human health and wellbeing. *S*. *aureus* is one of the pathogens that infects a wide range of hosts, and cause mild to serious illnesses. The propensity of *S*. *aureus* to build biofilm, makes them highly resistant to antimicrobials and host attack [27]. A holistic study was conducted in order to find effective biofilm inhibitors. Coumarin derivatives **1**–**21** were evaluated for potential antibiofilm activity using a colorimetric crystal violet assay [34]. The result showed that the antibiofilm effect of compounds **2**–**4**, **10**, and **17** overlapped with their antimicrobial effects, thus proving that the derivatives are potent biofilm inhibitors. We further investigated the disruption of biofilm by these derivatives through atomic force and fluorescence microscopy.

Discovery of new leads targeting distinct stages of biofilm formation, such as motility and EPS generation, adhesion, and virulence factors related to biofilm are of immense therapeutic significance. Biofilms are largely responsible of drug resistance [35]. This study was systematically conducted to explore the effects of synthetic compounds of various classes at biofilm formation by *S*. *aureus* ATCC 6538. We found that compounds belonging to coumarin class exhibited a potent anti-biofilm activity and also has low effect on the growth of bacteria. Coumarins are known for their therapeutic potential against different diseases. They possess the ability of blocking the quorum sensing signaling, and to inhibit biofilm formation in therapeutically relevant pathogens.

Out of coumarin 21 derivatives, compounds **2**, **3**, **4**, **10**, and **17** exhibited a low antibacterial effect on the growth of *S*. *aureus*, and high potential to inhibit the biofilms. Some compounds were found to have more antibacterial effect (compounds **9**, and **15**) or dual anti-bacterial and anti-biofilm effect (compounds **6**, **8**, **11**, and **14**). Compounds **2**–**4**, **10**, and **17** showed MBIC values ranging between 25, and 100 μg/mL. Compound **3** exhibited the highest anti-biofilm activity with MBIC value of 25 μg/mL, with low anti-bacterial effect.

Using AFM, we evaluated the effect of compounds on the EPS matrix. It has been reported that AFM is a key tool for assessing the topographic features of compound treated, and untreated microbial EPS matrix. The EPS matrix is a significant contributing virulence factor for the biofilm formation. The untreated control had a distinct, visible EPS network consisting of compact, irregularly dispersed lumps. The intra- and inter-molecular aggregation of polysaccharide macromolecules may be the cause of the lumps. Because of its high structural rigidity, EPS is used as a polymeric scaffold to build biofilms. No apparent pores, grooves, cell membrane ruptures, or bubbles in the untreated cells were observed. However, cells treated with the test compound displayed a possibly disturbed and deteriorated biofilm, with surface-level cellular debris. Additionally, there was lysis, blebbing, and cellular collapse with evident bulges and grooves. The cells were seen to be swollen with increased surface area. In addition to this, deep ruptures and roughness on biofilm surface, along with leakage of intracellular material and EPS matrix, was also observed. These compounds show a significant reduction in the height and surface roughness of *S*. *aureus* EPS.

Protease, DNase, quorum sensing, environmental cues, and other global regulatory mechanisms are all intricate parts of the genetic mechanism that drives biofilm formation in *S*. *aureus*. Downregulation of ica operon is a known mechanism in inhibiting the biofilm formation in *S*. *aureus* [36]. Compounds **2**–**4**, **10**, and **17** have significantly downregulated *icaA* and *icaD* genes when treated with active coumarin derivatives at their MBIC values. Hence, these results suggest that compounds **2**–**4**, **10**, and **17** mainly inhibited the biofilm formation by reducing the expressions of *icaA* and *icaD* genes. It can also be concluded from the present study that the coumarin derivatives exert its inhibitory effect on the adhesion phase of biofilm formation resulting in reduced biomass. Recent studies also suggest that *fnbA* and *fnbB* genes are strongly expressed only when the surface is covered with human fibronectin and they are positively influenced by the global regular *SarA* [37]. However, it is observed that fnb does not play a substantial role in biofilm formation in methicillin sensitive strains of *S*. *aureus* [38]. Further research is yet to be conducted in order to access the role of other genes involved directly and indirectly in the complex process of biofilm formation.

Compounds were also found to be non-cytotoxic. Their potential to inhibit the biofilm formation, and associated virulence factor (primarily EPS) in *S*. *aureus* is an effective approach in controlling biofilm related infections, caused by *S*. *aureus*. Development of antibiofilm agents is an important biomedical research [39]. This is imperative for delivering tangible solutions to highly prevalent biofilm related infections. This research thereby identified non cytotoxic compounds as leads for further research towards novel inhibitors /disruptors of biofilm formation.

## Conclusion

This study reveals that among all the coumarin derivatives synthesized, i.e. compounds **2–4**, **10**, and **17** were significantly able to inhibit biofilm formed by *S*. *aureus* (S1–S5 Tables). The results of scanning electron microscopy, fluorescence microscopy, and atomic force microscopy showed reduced biofilm development which was further confirmed with RT-PCR analysis, indicating down- regulation of *icaA*, and *icaD* genes which are primarily involved in the adhesion of cells to the surfaces (S6 and S7 Tables).

## Supporting information

S1 Table Percentage inhibition of compound 2 against *S*. *aureus* ATCC 6538. (DOCX)

S2 Table Percentage inhibition of compound 3 against *S*. *aureus* ATCC 6538. (DOCX)

S3 Table Percentage inhibition of compound 4 against *S*. *aureus* ATCC 6538. (DOCX)

S4 Table Percentage inhibition of compound 10 against *S*. *aureus* ATCC 6538. (DOCX)

S5 Table Percentage inhibition of compound 17 against *S*. *aureus* ATCC 6538. (DOCX)

S6 Table Ct values of *icaD* and reference gene in presence of compounds 2–4,10 and 17. (DOCX)

S7 Table Ct values of *icaA* and reference genes in presence of compounds 2–4,10 and 17. (DOCX)
