# Supplementary material for: Coumarin derivatives as new anti-biofilm agents against Staphylococcus aureus
Source: PLoS One. 2024 Sep 19;19(9):e0307439. doi: 10.1371/journal.pone.0307439 (PMC11412489; doi:10.1371/journal.pone.0307439)
Supplement: S1 Table — (DOCX) [file pone.0307439.s001.docx]

**Table-S1:** Percentage Inhibition of compound **2** against *S. aureus* ATCC 6538.

| **Compound 2** | | | | | | |
| --- | --- | --- | --- | --- | --- | --- |
| **Concentration µg/mL** | **% Inhibition 1** | **% Inhibition 2** | **% Inhibition 3** | **Mean % Inhibition** | **±SEM** | **SD** |
| 3.125 | 4.05 | 3.79 | 4.17 | 4.003333 | 0.590389 | 1.446151 |
| 6.25 | 3.29 | 3.99 | 3.49 | 3.59 | 14.71741 | 6.05015 |
| 12.5 | 1.73 | 0.99 | 2.54 | 1.753333 | 14.01274 | 4.324072 |
| 25 | 1.79 | 1.44 | 1.99 | 1.74 | 11.72478 | 2.719733 |
| 50 | 27.02 | 29.23 | 28.54 | 28.26333 | 6.218725 | 1.232703 |
| 100 | 95.01 | 93.91 | 93.59 | 94.17 | 3.244116 | 7.94643 |
