# Supplementary material for: Coumarin derivatives as new anti-biofilm agents against Staphylococcus aureus
Source: PLoS One. 2024 Sep 19;19(9):e0307439. doi: 10.1371/journal.pone.0307439 (PMC11412489; doi:10.1371/journal.pone.0307439)
Supplement: S2 Table — (DOCX) [file pone.0307439.s002.docx]

**Table-S2:** Percentage Inhibition of compound **3** against *S. aureus* ATCC 6538.

| **Compound 3** | | | | | | |
| --- | --- | --- | --- | --- | --- | --- |
| **Concentration µg/mL** | **% Inhibition 1** | **% Inhibition 2** | **% Inhibition 3** | **Mean % Inhibition** | **±SEM** | **SD** |
| 3.125 | 12.72 | 11.74 | 13.94 | 12.8 | 7.057291 | 7.286761 |
| 6.25 | 22.74 | 21.12 | 22.5 | 22.12 | 3.481127 | 8.526985 |
| 12.5 | 26.88 | 29.21 | 27.35 | 27.81333 | 12.83713 | 3.444417 |
| 25 | 55.92 | 57.84 | 56.64 | 56.8 | 6.314378 | 5.467004 |
| 50 | 73.99 | 75.95 | 76.99 | 75.64333 | 2.010432 | 4.924534 |
| 100 | 93.19 | 90.18 | 93.13 | 92.16667 | 1.072193 | 2.626325 |
