# Supplementary material for: Coumarin derivatives as new anti-biofilm agents against Staphylococcus aureus
Source: PLoS One. 2024 Sep 19;19(9):e0307439. doi: 10.1371/journal.pone.0307439 (PMC11412489; doi:10.1371/journal.pone.0307439)
Supplement: S3 Table — (DOCX) [file pone.0307439.s003.docx]

**Table-S3:** Percentage Inhibition of compound **4** against *S. aureus* ATCC 6538.

| **Compound 4** | | | | | | |
| --- | --- | --- | --- | --- | --- | --- |
| **Concentration µg/mL** | **% Inhibition 1** | **% Inhibition 2** | **% Inhibition 3** | **Mean % Inhibition** | **±SEM** | **SD** |
| 3.125 | 2.92 | 1.79 | 3.04 | 2.583333 | 13.08741 | 3.057469 |
| 6.25 | 7.04 | 8.05 | 9.02 | 8.036667 | 9.778903 | 5.953321 |
| 12.5 | 6.69 | 5.79 | 5.68 | 6.053333 | 12.21347 | 4.916761 |
| 25 | 29.15 | 30.07 | 28.05 | 29.09 | 8.915822 | 2.839215 |
| 50 | 66.01 | 67.02 | 66.09 | 66.37333 | 9.303285 | 4.7883 |
| 100 | 86.79 | 84.34 | 86.62 | 85.91667 | 5.756752 | 5.101104 |
