# Supplementary material for: Coumarin derivatives as new anti-biofilm agents against Staphylococcus aureus
Source: PLoS One. 2024 Sep 19;19(9):e0307439. doi: 10.1371/journal.pone.0307439 (PMC11412489; doi:10.1371/journal.pone.0307439)
Supplement: S4 Table — (DOCX) [file pone.0307439.s004.docx]

**Table-S4:** Percentage Inhibition of compound 10 against *S. aureus* ATCC 6538.

| **Compound 10** | | | | | | |
| --- | --- | --- | --- | --- | --- | --- |
| **Concentration µg/mL** | **% Inhibition 1** | **% Inhibition 2** | **% Inhibition 3** | **Mean % Inhibition** | **±SEM** | **SD** |
| 3.125 | 1.98 | 1.44 | 1.54 | 1.653333 | 1.326115 | 3.248306 |
| 6.25 | 5.34 | 4.99 | 5.53 | 5.286667 | 40.39058 | 4.936311 |
| 12.5 | 7.559 | 7.29 | 7.21 | 7.353 | 33.017 | 8.874791 |
| 25 | 2.34 | 2.21 | 2.09 | 2.213333 | 35.21448 | 6.257499 |
| 50 | 39.49 | 39.76 | 39.78 | 39.67667 | 34.74653 | 5.111269 |
| 100 | 69.39 | 69.52 | 68.9 | 69.27 | 9.340172 | 2.878656 |
