# Supplementary material for: Coumarin derivatives as new anti-biofilm agents against Staphylococcus aureus
Source: PLoS One. 2024 Sep 19;19(9):e0307439. doi: 10.1371/journal.pone.0307439 (PMC11412489; doi:10.1371/journal.pone.0307439)
Supplement: S5 Table — (DOCX) [file pone.0307439.s005.docx]

**Table-S5:** Percentage Inhibition of compound **17** against *S. aureus* ATCC 6538.

| **Compound 17** | | | | | | |
| --- | --- | --- | --- | --- | --- | --- |
| **Concentration µg/mL** | **% Inhibition 1** | **% Inhibition 2** | **% Inhibition 3** | **Mean % Inhibition** | **±SEM** | **SD** |
| 3.125 | 1.638 | 1.356 | 1.843 | 1.612333 | 1.081676 | 2.649553 |
| 6.25 | 3.64 | 3.52 | 3.97 | 3.71 | 11.61703 | 8.455806 |
| 12.5 | 0.03 | 0.03 | 0.01 | 0.023333 | 11.91768 | 9.192245 |
| 25 | 15.854 | 15.05 | 16.86 | 15.92133 | 6.781021 | 6.61004 |
| 50 | 55.073 | 52.997 | 57 | 55.02333 | 5.681644 | 3.917129 |
| 100 | 77.89 | 77.63 | 82.34 | 79.28667 | 1.759374 | 4.309568 |
