# Supplementary material for: Coumarin derivatives as new anti-biofilm agents against Staphylococcus aureus
Source: PLoS One. 2024 Sep 19;19(9):e0307439. doi: 10.1371/journal.pone.0307439 (PMC11412489; doi:10.1371/journal.pone.0307439)
Supplement: S6 Table — (DOCX) [file pone.0307439.s006.docx]

**Table-S6:** Ct values of *icaD* and reference gene in presence of compounds **2-4**,**10** and **17.**

**q-PCR Analysis of *icaA* and *icaD***

| **Compound Number** | **Concentration µg/mL** | **Reference Gene** | **Ct** | **Target Gene** | **Ct** |
| --- | --- | --- | --- | --- | --- |
| **2** | 100 | 16S rRNA | 31.37 | *icaD* | 17.32 |
| **3** | 25 | 16S rRNA | 31.67 | *icaD* | 19.34 |
| **4** | 50 | 16S rRNA | 31.52 | *icaD* | 18.61 |
| **10** | 100 | 16S rRNA | 31.92 | *icaD* | 18.99 |
| **17** | 100 | 16S rRNA | 30.78 | *icaD* | 17.09 |
