# Supplementary material for: Coumarin derivatives as new anti-biofilm agents against Staphylococcus aureus
Source: PLoS One. 2024 Sep 19;19(9):e0307439. doi: 10.1371/journal.pone.0307439 (PMC11412489; doi:10.1371/journal.pone.0307439)
Supplement: S7 Table — (DOCX) [file pone.0307439.s007.docx]

| **Compound Number** | **Concentration µg/mL** | **Reference Gene** | **Ct** | **Target Gene** | **Ct** |
| --- | --- | --- | --- | --- | --- |
| **2** | 100 | 16S rRNA | 31.37 | *icaA* | 17.32 |
| **3** | 25 | 16S rRNA | 31.67 | *icaA* | 18.35 |
| **4** | 50 | 16S rRNA | 31.52 | *icaA* | 19.21 |
| **10** | 100 | 16S rRNA | 31.92 | *icaA* | 18.59 |
| **17** | 100 | 16S rRNA | 30.78 | *icaA* | 17.33 |

**Table-S7:** Ct values of *icaA* and reference genes in presence of compounds **2-4**,**10** and **17**.
